# Real-time PCR for differential quantification of CVI988 vaccine virus and virulent strains of Marek’s disease virus

**DOI:** 10.1016/j.jviromet.2016.03.002

**Published:** 2016-07

**Authors:** Susan J. Baigent, Venugopal K. Nair, Hervé Le Galludec

**Affiliations:** a Avian Oncogenic Virus Group, The Pirbright Institute, Woking, GU24 0NF, United Kingdom; b Zoetis International Services, 23-25 Avenue du Docteur Lannelongue, 75668 Paris Cedex 14, France

**Keywords:** 40-Ct, cycle threshold value in real-time PCR subtracted from 40, BAC, bacterial-artificial-chromosome, dpc, days post challenge, dpv, days post vaccination, FAM-BHQ1, probe with carboxyfluorescein (FAM) reporter fluorochrome and Black Hole Quencher, LOD, limit of detection by q-PCR, ovo, chicken ovotransferrin gene, PBL, peripheral blood lymphocytes, pp38-CVI, PCR probe specific for CVI988 pp38 gene sequence, pp38-Vir, PCR probe specific for virulent MDV pp38 gene sequence, q-PCR, real-time quantitative polymerase chain reaction, SNP, single nucleotide polymorphism, YY-TAMRA, probe with Yakima Yellow reporter fluorochrome and tetramethylrhodamine quencher, Marek’s disease virus, Vaccination, CVI988/Rispens, Real-time PCR, pp38, Single nucleotide polymorphism

## Abstract

CVI988/Rispens vaccine, the ‘gold standard’ vaccine against Marek’s disease in poultry, is not easily distinguishable from virulent strains of Marek’s disease herpesvirus (MDV). Accurate differential measurement of CVI988 and virulent MDV is commercially important to confirm successful vaccination, to diagnose Marek’s disease, and to investigate causes of vaccine failure. A real-time quantitative PCR assay to distinguish CVI988 and virulent MDV based on a consistent single nucleotide polymorphism in the pp38 gene, was developed, optimised and validated using common primers to amplify both viruses, but differential detection of PCR products using two short probes specific for either CVI988 or virulent MDV. Both probes showed perfect specificity for three commercial preparations of CVI988 and 12 virulent MDV strains. Validation against BAC-sequence-specific and U_S_2-sequence-specific q-PCR, on spleen samples from experimental chickens co-infected with BAC-cloned pCVI988 and wild-type virulent MDV, demonstrated that CVI988 and virulent MDV could be quantified very accurately. The assay was then used to follow kinetics of replication of commercial CVI988 and virulent MDV in feather tips and blood of vaccinated and challenged experimental chickens. The assay is a great improvement in enabling accurate differential quantification of CVI988 and virulent MDV over a biologically relevant range of virus levels.

## Highlights

• Developed, optimised and validated q-PCR to distinguish CVI988 and virulent MDV.
• Based on differential detection of single nucleotide polymorphism in pp38 gene.
• Specific, sensitive, efficient, reproducible assay with no false-positive results.
• Accurate differential quantification over biological range of virus levels.
• Replication of CVI988 and virulent MDV examined in chicken feathers and blood.

## 1 Introduction

Marek’s disease (MD) is a highly infectious lymphoid neoplasm of chickens caused by oncogenic serotype-1 strains (MDV-1) of Marek’s disease virus (family *Herpesviridae,* subfamily *Alphaherpesvirinae,* genus *Mardivirus*). Susceptible breeds of chicken develop visceral and neural lymphomatous lesions (Baigent and Davison, 2004, Calnek, 2001), resulting in death or carcass condemnation. This economically important disease has been successfully controlled since the 1970s by vaccination using cell-associated live avirulent and nononcogenic vaccine strains of all three *Mardivirus* serotypes. Serotype 2 (MDV-2) or serotype 3 (herpesvirus of turkeys, HVT) vaccine viruses are naturally avirulent in chickens (Okazaki et al., 1970, Schat and Calnek, 1978), while CVI988/Rispens vaccine (de Boer et al., 1986, Rispens et al., 1972) is a naturally-attenuated MDV-1 strain. MD vaccine viruses establish persistent infection and lifelong immunity, effectively protecting against tumours and mortality. However, vaccination does not prevent superinfection, replication and shedding of virulent challenge viruses (Davison and Nair, 2005, Gimeno, 2008), so chickens can potentially be infected simultaneously with both vaccine and virulent MDV strains.

Accurate differential measurement of vaccine and virulent virus in the same individual chicken is both commercially important and experimentally useful. In the field, it could confirm successful vaccination and assist in identifying causes of vaccine failure, such as administration of a sub-optimal vaccine dose (Landman and Verschuren, 2003), interference with vaccine virus replication by maternal antibodies (King et al., 1981, de Boer et al., 1986), and infection with virulent MDV field strains (Witter et al., 2005). In the laboratory, it could be used to study mechanisms of vaccinal protection and differences in efficacy between vaccines.

Therefore, it is important to have a reliable test for differentiation of MDV-infected and vaccinated animals (DIVA test). Real-time quantitative polymerase chain reaction (q-PCR) is a sensitive and specific molecular diagnostic method. Serotype-specific q-PCR can be used to distinguish and differentially quantify MD vaccine and challenge virus, where vaccine is of serotype 2 (Islam et al., 2004, Renz et al., 2006) or serotype 3 (Islam et al., 2004, Islam et al., 2006a, Islam et al., 2006b), and thus of a different serotype to challenge virus (always serotype 1). CVI988 is the ‘gold standard’ vaccine against MD because it is antigenically and genetically 98% identical to virulent strains (Spatz et al., 2007a), and therefore elicits a very effective immune response. However, the downside of this is that CVI988 is not easily distinguishable from virulent MDV-1, restricting the application of differential molecular diagnostics (Baigent et al., 2006b). To date, development of a q-PCR assay which is both accurately quantitative and able to universally distinguish all virulent MDV strains from CVI988, has not been possible.

Using bacterial-artificial-chromosome (BAC) cloned CVI988 virus (pCVI988), which carries the BAC vector sequences in place of the MDV-1 U_S_2 gene, q-PCR assays for differential quantification of pCVI988 from virulent MDV (by targeting the BAC vector sequence and the U_S_2 gene respectively) were developed previously (Baigent et al., 2011). Although these assays allowed investigation of replication of both pCVI988 vaccine virus and virulent MDV-1 in experimental infections (Baigent et al., 2011), they cannot be used in the field as commercial CVI988 does not contain a BAC sequence. However, the BAC-U_S_2 q-PCR system, and the DNA samples generated in that study, represents an ideal system against which to validate any prospective CVI988-specific and virulent MDV-1-specific q-PCR assays.

Spatz and Silva (2007) identified several types of sequence difference in the repeat long (RL) genomic regions of 13 MDV-1 strains of varying virulence: polynucleotide sequence expansions, short tandem repeat polymorphisms, sequence deletions, frameshift mutations and single nucleotide polymorphisms (SNPs). The efforts of other researchers to develop DIVA PCR assays for CVI988 and virulent MDV have focused on the polynucleotide sequence expansion of the 132-bp repeat region, the 177-bp insertion of duplicated sequence in the CVI988 *meq* gene, and SNPs in the pp38 gene. Conventional PCR to amplify the 132-bp repeat region (Becker et al., 1992, Silva, 1992) could distinguish CVI988 from virulent MDVs based on a difference in the number of 132-bp repeats, but had relatively low sensitivity (Davidson et al., 2002). A highly sensitive quantitative *meq* gene q-PCR, that detects both CVI988 and virulent MDV (Baigent et al., 2005a), can be used to quantify either CVI988 or virulent MDV if only one of those viruses is present (Baigent et al., 2005a, Baigent et al., 2005b, Baigent et al., 2006a, Baigent et al., 2006b), or can be paired with the 132-bp repeat PCR to assist with distinction between CVI988 and virulent MDV in mixed infections. However, 132-bp repeat PCR is not very sensitive for CVI988 (since the multiple bands of the CVI988 PCR products have much lower intensity than the single/double bands of the virulent MDV PCR products), not absolutely conclusive (Baigent et al., 2005b, van Iddekinge et al., 1999, Niikura et al., 2006, Petherbridge et al., 2003, Silva and Gimeno, 2007), laborious, and the repetitive nature of the 132-bp repeat region precludes its use as a quantitative assay.

The *meq* gene is polymorphic, with variation in the number of sequence repeats in the proline-rich region (Chang et al., 2002). Murata et al. (2007) developed a non-quantitative nested PCR to distinguish between standard *meq* and large (L-)*meq* (with a 177-bp insertion). However, while virulent MDV strains have standard *meq*, CVI988 may have either L-*meq* or standard *meq* (Lee et al., 2000), so the 177-bp insertion is not a reliable differential marker for CVI988 and virulent MDV. Based on a stable polymorphism in the *meq* gene, Renz et al. (2013) developed two sensitive and specific real-time PCRs to differentiate and accurately quantify 20 Australian virulent MDV isolates and three commercial CVI988 vaccines used in Australia. However, virulent isolates from USA, Asia and Europe do not differ from CVI988 at this polymorphism so, while this assay could be very successfully used in Australia, it could not be used globally (Renz et al., 2013).

The MDV-1 pp38 gene has a SNP at base #320, which is consistent between CVI988 (which has ‘G’ at this position) and all sequenced virulent strains (which have ‘A’) (Cui et al., 1991, Endoh et al., 1994, Spatz et al., 2007a, Spatz et al., 2007b). The current study therefore investigated the possibility of using SNP #320 as a reliable biomarker for differentiating and quantifying CVI988 and virulent strains by real-time PCR (pp38 SNP q-PCR assay). Subsequent to development of this assay, Gimeno et al. (2014) published a real-time PCR assay also based on SNP #320. However, their assay differs from the assay reported in the current paper in terms of the molecular methods used to amplify and detect the polymorphism, their ability to fully validate the assay, and its quantitative accuracy.

The aims of the current work were (1) to design and optimise a q-PCR assay based on SNP #320 in the pp38 gene to specifically distinguish CVI988 from virulent MDV-1 strains; (2) to investigate the feasibility of making this assay accurately quantitative over a biologically relevant range of virus levels; (3) to validate the assay against the BAC/U_S_2 q-PCR assay; and (4) to use the assay to investigate replication of CVI988 and virulent MDV in tissues of vaccinated, challenged experimental chickens.

## 2 Materials and methods

### 2.1 Design of q-PCR primers and probes

**Fig. 1:**
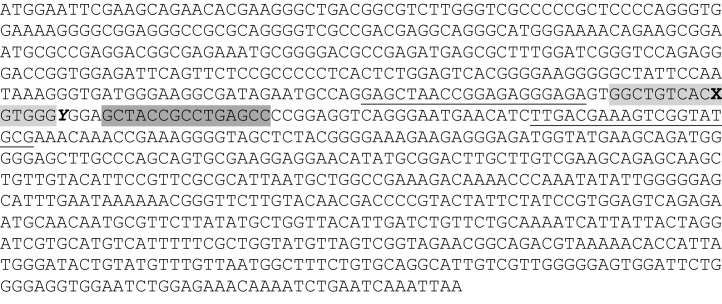
Location of primers and probes in MDV-1 pp38 gene sequences. The pp38 gene sequence shows two single nucleotide differences (G/A SNPs) between CVI988 and Md5 (a representative virulent strain). The bold SNP X (#320) represents a consistent difference between CVI988 and virulent strains. The bold italicised SNP Y (#326) is not consistent in all virulent strains. Sequences of the primers (underlined text) were: forward primer 5′-GAGCTAACCGGAGAGGGAGA-3′, and reverse primer 5′-CGCATACCGACTTTCGTCAA-3′, and the amplicon size was 99 bp. Two 15-mer probes (light grey shading), designed to the minus strand, covered the region containing SNP #320: pp38-CVI (5′-CCCAC**C**GTGACAGCC-3′, Tm = 57.7) and pp38-Vir(1) (5′-CCCAC**T**GTGACAGCC-3′, Tm = 54.1). A third (17-mer) probe, pp38-Vir(3) included both SNPs #320 and #326 (5′-C**T**CCCAC**T**GTGACAGCC-3′, Tm = 57.1). A fourth probe (pp38-Generic, 5′-GCTACCGCCTGAGCC-3′, designed to the plus strand, Tm = 57.4) which targets a region common to pp38 of all MDV-1 strains, is shaded dark grey.

Primers and probes were designed by AlleLogic Biosciences Corp (Hayward, CA). Primer and TaqMan^®^ probe sequences, and their location within the pp38 gene, are shown in Fig. 1. The forward and reverse primers (pp38-FP and pp38-RP) are common to both the CVI988 and virulent MDV-1 pp38 sequences, and the 99-bp amplicon covers the polymorphic region within which the probes were designed. One 15-mer probe (pp38-Generic) was designed to target a region common to all MDV-1 strains. Two 15-mer probes were designed to incorporate the consistent G/A SNP #320, one specific for the CVI988 sequence (pp38-CVI), and one specific for the virulent MDV-1 sequence (pp38-Vir(1)). Subsequently, an additional probe (pp38-Vir(3)) was designed for detection of virulent MDV-1, to have a melting temperature similar to that of the pp38-CVI probe and the pp38-Generic probe. Probe pp38-Vir(3) also incorporates the ‘non-consistent’ SNP #326, so it is possible that affinity for some field strains may be reduced. However, in this study, the specificity of pp38-Vir(1) and pp38-Vir(3) was identical, but pp38-Vir(3) was chosen as the probe for routine detection of virulent MDV-1 because it was more sensitive. Each probe was labelled with 5′FAM reporter, and 3′BHQ1 quencher. All probes were used in combination with the same primer pair. All primers and probes were manufactured by Sigma Genosys (UK).

### 2.2 DNA samples from virus stocks and chicken tissues

HVT strain FC126, MDV-1 strain HPRS-B14, and USA MDV-1 strains 675A, 584A, 648A, 660A, 595, Md5, 549, 571, JM102/W (Witter, 1997, Witter et al., 2005) were obtained from Dr. A.M. Fadly (Avian Disease and Oncology Laboratory, USA) all as 7th duck embryo fibroblast passage stocks. MDV-1 strain RB-1B was prepared as previously described (Baigent et al., 2007). European MDV-1 isolate C12/130 (Barrow and Venugopal, 1999) was prepared as previously described (Smith et al., 2011). Three commercial CVI988 vaccine stocks were used: Pfizer Poulvac Marek CVI, Merial BioMarekR, and Intervet Nobilis Rismavac. MDV-2 strain SB-1 was a commercial vaccine stock (SB-vacc, Intervet). pCVI988 virus was prepared from the BAC clone of Poulvac CVI988 DNA, as previously described (Petherbridge et al., 2003). All viruses were prepared as cell-associated stocks grown in chicken embryo fibroblast cells (CEF). For each virus stock, DNA was prepared from approximately 5 × 10^6^ cells using the DNeasy-96 kit (Qiagen), according to the manufacturer’s instructions. Chicken tissue DNA samples were available from a previous study from experimental chickens vaccinated with pCVI988 and/or challenged with virulent strain RB-1B (Baigent et al., 2011).

### 2.3 Real-time quantitative PCR

Reactions were set up on ice, in 96-well plates in a PCR-dedicated cabinet, using PCR-dedicated pipettes and aerosol resistant autoclaved tips. A master-mix was prepared diluting the components in water, and 21 μl aliquotted per reaction, so that each reaction contained: primers pp38-FP and pp38-RP (0.4 μM), one of the above pp38 probes (0.2 μM) and ABsolute Blue^®^ q-PCR Low Rox master-mix (Thermofisher UK). Duplex reactions to detect both pp38 and the chicken ovotransferrin (*ovo*) gene also contained *ovo* forward and reverse primers (0.4 μM), and *ovo* probe (0.2 μM, 5′Yakima Yellow-3′TAMRA, Eurogentec), the sequences of which were reported previously (Baigent et al., 2005a). All reactions were made up to 25 μl by the addition of 4 μl water (‘no template control’ wells), test DNA, standard DNA or control DNA. Every run included RB-1B DNA control (positive control for virulent MDV detection and negative control for CVI988 detection) and CVI988 DNA control (positive control for CVI988 detection and negative control for virulent MDV detection) to ensure correct setting of the threshold. q-PCRs targeting the BAC and U_S_2 gene sequences were performed as previously described (Baigent et al., 2011). Standard curves were prepared using 10-fold serial dilutions of DNA from RB-1B-infected CEF (for pp38-Vir reaction and for U_S_2 reaction), pCVI988-infected CEF (for pp38-CVI reaction and for BAC reaction) and non-infected CEF (for *ovo* reaction) all of which had been accurately calibrated against plasmid constructs of known target gene copy number: pRB-1B (Petherbridge et al., 2004), pCVI988 (Petherbridge et al., 2003), and pGEMT-ovo (Baigent et al., 2005a). An ABI7500^®^ system (Applied Biosystems) was used to amplify and detect the reaction products, under the following conditions: 95 °C for 15 min, followed by 40 cycles of 95 °C (15 s) and 60 °C (1 min). For chicken tissue DNA samples, MDV genomes were normalised to 2 × 10^4^ copies of the chicken *ovo* gene, so the standard curves enabled quantification of each virus as genomes per 10^4^ cells (Baigent et al., 2005a).

### 2.4 Quantitative accuracy of detection in virus mixtures.

Testing of sensitivity and quantitative accuracy of the pp38 q-PCRs, in mixed samples over a wide range of CVI988:virulent MDV ratios, was performed and validated against BAC-specific and U_S_2-specific q-PCRs, as described below. DNA prepared from CEF infected with pCVI988 virus, and DNA prepared from CEF infected with RB-1B was used to prepare dilution series. The starting concentration of each DNA sample was approximately 5 × 10^5^ genomes per 4 μl. DNA of pCVI988 was diluted in DNA of RB-1B or in DNA from non-infected CEF, in two parallel dilution series; and DNA of RB-1B was diluted in DNA of pCVI988 or in DNA from non-infected CEF, in two parallel dilution series. Dilutions were 1:2, 1:5, 1:10, 1:30, 1:10^2^, 1:10^3^ and 1:10^4^, each in duplicate. The pp38 SNP q-PCR was validated by two means: (1) Comparing measurements from samples diluted in DNA from the other virus with measurements from samples diluted in chicken DNA from non-infected CEF; (2) Comparing measurements made by the pp38-CVI q-PCR and the pp38-Vir q-PCR, with those made using BAC-specific and U_S_2-specific q-PCRs respectively.

### 2.5 Validation of pp38 q-PCR assays on chicken tissue samples

Spleen DNA samples from experimental chickens which had been vaccinated with BAC-cloned CVI988 virus (pCVI988) and/or challenged with RB-1B, were available from a previous study (Baigent et al., 2011). In that study, pCVI988 vaccine virus and RB-1B challenge virus were distinguished and specifically quantified using TaqMan^®^ q-PCR to measure a BAC-specific target sequence, and the U_S_2 gene target sequence, respectively. In the current work, the spleen DNA samples from this study were subjected to pp38 SNP/*ovo* duplex q-PCR assays, using either pp38-CVI, pp38-Vir(3), or pp38-Generic as the pp38 detection probe. The appropriate standard curves were used to calculate genome copy no. per 10^4^ cells for CVI988, RB-1B or total MDV-1, respectively.

### 2.6 Time-course of replication of commercial CVI988 vaccine and RB-1B challenge virus in individual chickens

Thirty-two one-day-old specified pathogen-free, maternal-antibody-free Rhode Island Red chickens were randomly allocated to three experimental groups. All procedures were performed according to the guidelines of the UK Home Office. The study was performed under a Project Licence issued by the UK Home Office and approved by the Ethical Review Committee at The Pirbright Institute. Vaccination and challenge were performed only by trained persons in possession of a Personal Home Office Licence permitting them to perform these procedures.

Chickens were vaccinated by subcutaneous administration of one commercial dose of Poulvac CVI988 (Pfizer Animal Health) at one day of age. Chickens were challenged at 8 days of age, by intra-abdominal administration of 100 μl dose containing 1000 pfu MDV strain RB-1B (Schat et al., 1982) prepared as previously described (Baigent et al., 2007). Group A (n = 8) received vaccine only, Group B (n = 14) received challenge virus only, and Group C (n = 10) was both vaccinated and challenged. Group A initially had 10 chickens, but two were ‘non-starters’ and were culled prior to challenge. The greater number of chickens in Group B was intentional, since it was expected that some would die in the first two weeks post challenge. The three groups were housed in separate animal rooms which were high efficiency particulate air-filtered and maintained at negative pressure. Every chicken was sampled by trained personnel at ten time-points between -4 and 38 days post challenge (dpc), equivalent to 3 and 45 days post vaccination (dpv). Peripheral blood lymphocyte (PBL) and feather tip samples were collected and prepared as previously described (Baigent et al., 2005a, Baigent et al., 2005b). Feather tips were collected from 10 dpv (3 dpc), the first time-point at which feathers were present in sufficient size. DNA was prepared from PBL and feather tips using the DNeasy-96 kit (Qiagen), according to the manufacturer’s instructions, and each DNA sample was subjected to pp38 SNP/*ovo* duplex q-PCR assays, using either pp38-CVI or pp38-Vir(3) as the pp38 detection probe. The appropriate standard curves were used to calculate genome copy no. per 10^4^ cells for CVI988 or RB-1B. Chickens were monitored daily for morbidity and mortality, and any chicken reaching the humane end-point was culled using a Schedule 1 method by trained persons. All surviving chickens were culled at 46 dpv (39 dpc). *Post mortem* examination was performed on every chicken.

### 2.7 Statistical analyses

Statistical analyses were performed using Minitab v15. To validate the efficacy of the pp38 SNP q-PCRs in quantifying CVI988 and RB-1B in virus mixtures, mean 40-Ct values for virus DNA diluted in non-infected CEF DNA were compared with mean 40-Ct values for virus DNA diluted in DNA of CEF infected with the other virus, using analysis of variance. To investigate correlation between genome copies calculated using the two q-PCR systems (BAC-U_S_2 q-PCR vs pp38 SNP q-PCRs), samples were compared in pairwise combinations using regression analysis. To examine the effect of co-infection on replication of CVI988 vaccine and RB-1B challenge viruses in PBL and feather tips, arithmetic mean values for virus genome copy number and 95% confidence intervals were determined using the log_10_ transformed copy number for each individual sample, then back-transformed to obtain actual values for presentation of results. Analysis of variance was performed to examine the effect of challenge with RB-1B on the level of CVI988 per 10^4^ cells in chickens from the two vaccinated groups, and the effect of vaccination on the level of RB-1B virus per 10^4^ cells in chickens from the two challenged groups. This analysis was performed within each sampling time-point (not as a repeated measures analysis), so sampling time was excluded as a factor in the model. Pairwise comparisons were made using Tukey simultaneous tests.

## 3 Results

### 3.1 Specificity of CVI988-specific and virulent MDV-1-specific pp38 probes in singleplex reactions

**Fig. 2:**
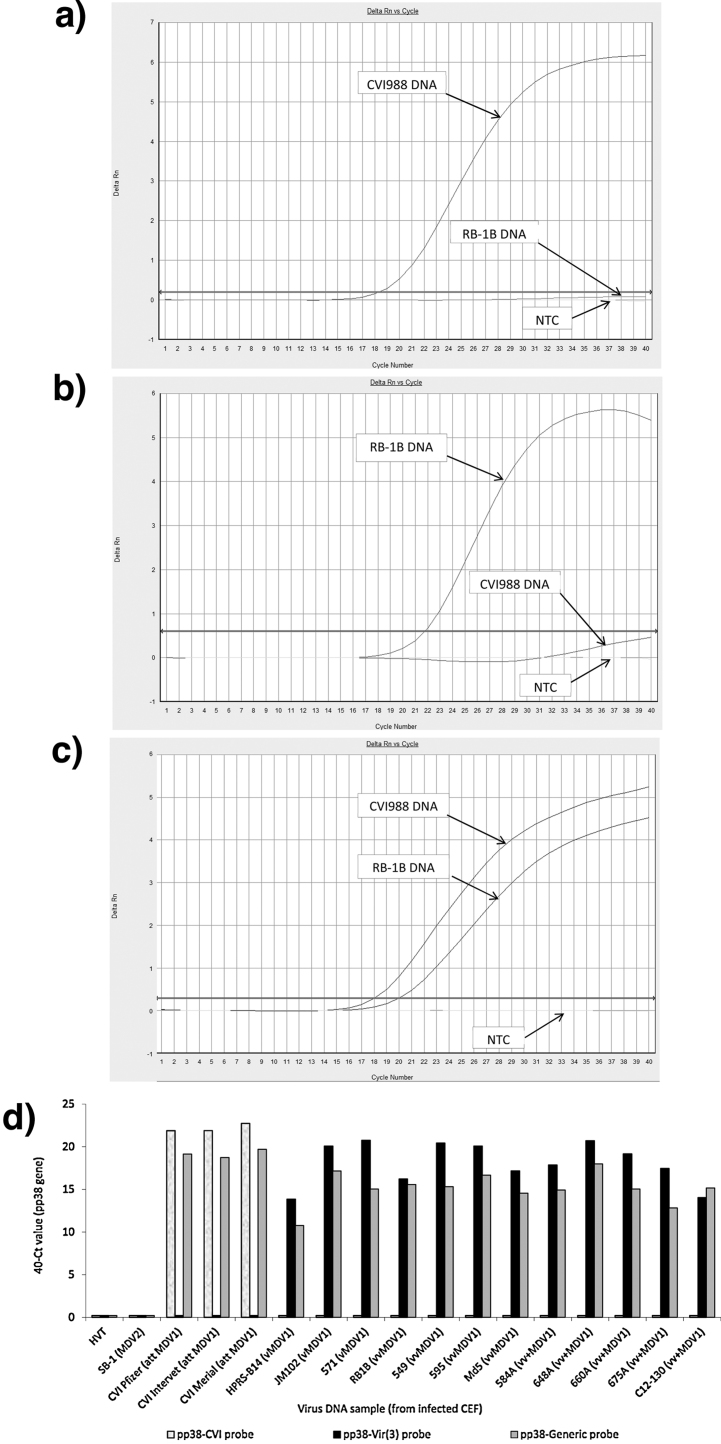
Specificity of probes for CVI988 and virulent MDV-1. Change in fluorescence (delta Rn) when pp38-CVI, pp38-Vir(3) and pp38-Generic probes were used in 40-cycle q-PCR using 100 ng DNA from either CVI988-infected CEF or RB-1B-infected CEF as a template. The thick horizontal line with an arrowhead at each end is the threshold (default value 0.2). (a) pp38-CVI probe detects CVI988 DNA but not RB-1B DNA; NTC is no-template control. (b) pp38-Vir(3) probe detects RB-1B DNA, while the signal from the CVI988 PCR product is very low. By raising the threshold line to 0.6, CVI988 is scored negative in this reaction. (c) pp38-Generic probe detects both CVI988 DNA and RB-1B DNA. (d) The specificities of pp38-CVI, pp38Vir-A(3) and pp38-Generic probes were tested against a range of Mardivirus strains in a 40-cycle q-PCR. The 40-Ct value (the cycle threshold value, Ct, subtracted from 40) is proportional to the amount of target DNA present. A 40-Ct value of 0 indicates that fluorescence did not cross the threshold, i.e. the probe did not bind to the PCR product. ‘att MDV1′ = attenuated MDV-1, vMDV1 = virulent MDV-1, vvMDV1 = very virulent MDV-1, vv+MDV1 = very virulent plus MDV-1.

Fig. 2 shows the change in fluorescence when pp38-CVI, pp38-Vir(3) and pp38-Generic probes were used in 40-cycle q-PCR using 100 ng DNA from either CVI988-infected CEF or RB-1B-infected CEF as a template. The pp38-CVI probe gave a large increase in fluorescence with CVI988 DNA template, while increase in fluorescence with RB-1B DNA template was minimal (fluorescence did not cross the threshold line at the default setting of 0.2, thus the sample is scored negative) (Fig. 2a). The pp38-Vir(3) probe gave a large increase in fluorescence with RB-1B DNA, and some increase with CVI988 DNA (Fig. 2b). Raising the threshold to 0.6 ensured that CVI988 was always scored negative in this assay and prevented false positive results. The pp38-Generic probe gave a large increase in fluorescence with both RB-1B and CVI988, with a threshold setting of 0.2 (Fig. 2c). None of the three probes gave any increase in fluorescence in no-template-control wells.

The specificity of the two probes was then tested against target DNA samples from a range of Mardiviruses: HVT (serotype 3), SB-1 (serotype 2), CVI988 vaccine from three different commercial sources, and 12 virulent MDV-1 strains covering the spectrum of virulence from virulent (v), very virulent (vv) to very virulent plus (vv+) (Witter et al., 2005). Fig. 2d shows that, using a detection threshold of 0.2 for pp38-CVI, this probe detected all three commercial CVI988 preparations, but did not detect any of the virulent MDV-1 strains, or HVT, or SB-1. Using a detection threshold of 0.6 for pp38-Vir(3), this probe detected all virulent MDV-1 strains, but did not detect CVI988, HVT or SB-1. Using a detection threshold of 0.2, pp38-Generic probe detected all CVI988 and all virulent strains of MDV-1, but did not detect HVT or SB-1.

### 3.2 Assay sensitivity, efficiency, linearity and reproducibility

**Fig. 3:**
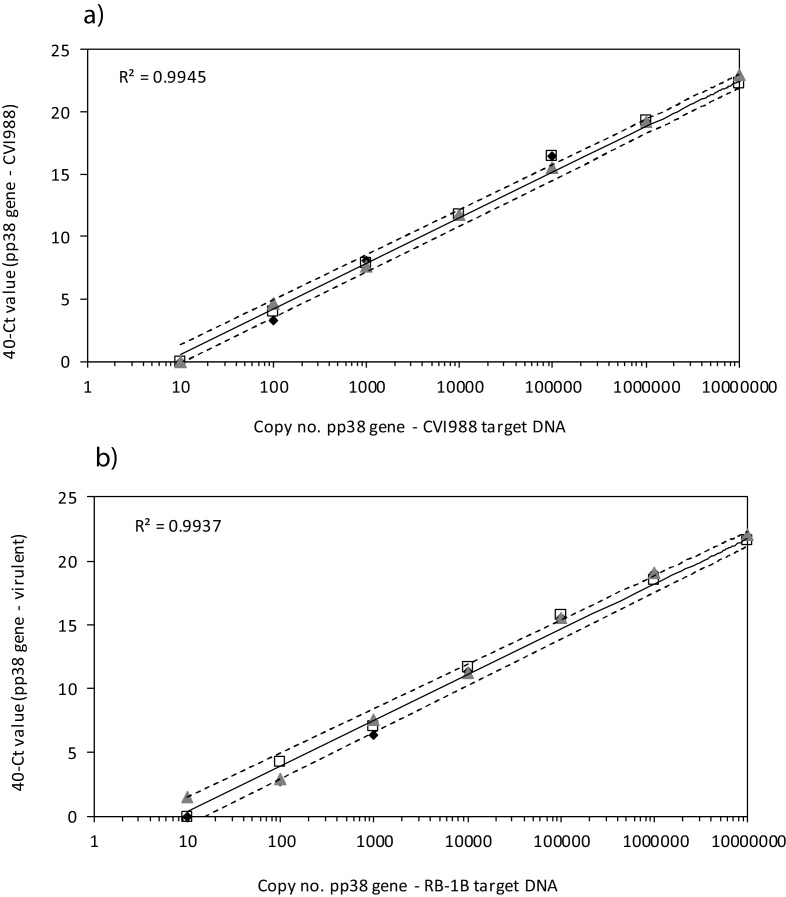
Assay sensitivity, efficiency, linearity and reproducibility. The probes were tested in q-PCR assays using serial dilutions of DNA extracted from CVI988-infected CEF or RB-1B-infected CEF. Mean 40-Ct values from triplicate assays, with 95% confidence limits for the regression and the R^2^ value, are shown for (a) pp38-CVI probe with serial dilutions of CVI988CEF DNA, and (b) pp38-Vir(3) probe with serial dilutions of RB-1B CEF DNA. The underlying points for 40-Ct values from triplicate assays are shown using three different symbols to distinguish the three assays (open squares, black diamonds and grey triangles).

The probes were tested in q-PCR assays using 10-fold serial dilutions of DNA prepared from CEF infected with either RB-1B or CVI988. Mean amplification efficiencies, R^2^ values and limits of detection (LOD) are summarised in Table 1. LOD is the value for x (gene copy no.) when y (40-Ct value) = 0. Fig. 3 shows the reproducibility of standard curves, across three independent q-PCR runs, using pp38-CVI probe with serial dilutions of CVI988 DNA (Fig. 3a) and pp38-Vir(3) probe with serial dilutions of RB-1B DNA (Fig. 3b). The assays show good reproducibility, sensitivity, linearity, efficiency, and a wide dynamic range.

### 3.3 Duplex q-PCR for absolute quantification of CVI988 and virulent MDV-1

In order to use the pp38 SNP q-PCR assays for absolute quantification of vaccine and challenge virus in tissue samples (expressed as virus genomes per 10^4^ cells), it was necessary to use duplex reactions to simultaneously measure either the CVI988 pp38 gene and the *ovo* gene, or the virulent MDV-1 pp38 gene and the *ovo* gene. The *ovo* probe was labelled with YY-TAMRA so as to be detected in a different channel to the pp38 probe fluorescence. The *ovo* and pp38 reactions were shown to proceed with the same efficiency when used in duplex reactions as in singleplex reactions (data not shown). The mean amplification efficiency, R^2^ value and LOD for the *ovo* reaction are shown in Table 1.

### 3.4 Quantitative accuracy of detection in virus mixtures

Since the pp38 SNP q-PCR assay uses a universal primer pair, complementary to a conserved region of the pp38 gene, both CVI988 and virulent strains of MDV will be amplified from a mixed population, and thus there is no reaction specificity at this level (specificity is determined solely by probe binding). Therefore, when both CVI988 and virulent MDV target sequences are present, they will compete for the same primers. If one virus is present at a high level, and the other at only a low level, it is possible that competition for primers would prevent amplification (and thus detection) of the less abundant virus. The ability of the reactions to accurately quantify both CVI988 and virulent MDV-1 in mixed samples was examined and validated against BAC-specific and U_S_2-specific q-PCRs (which will distinguish cloned pCVI988 and RB-1B without any competition for reagents).

**Fig. 4:**
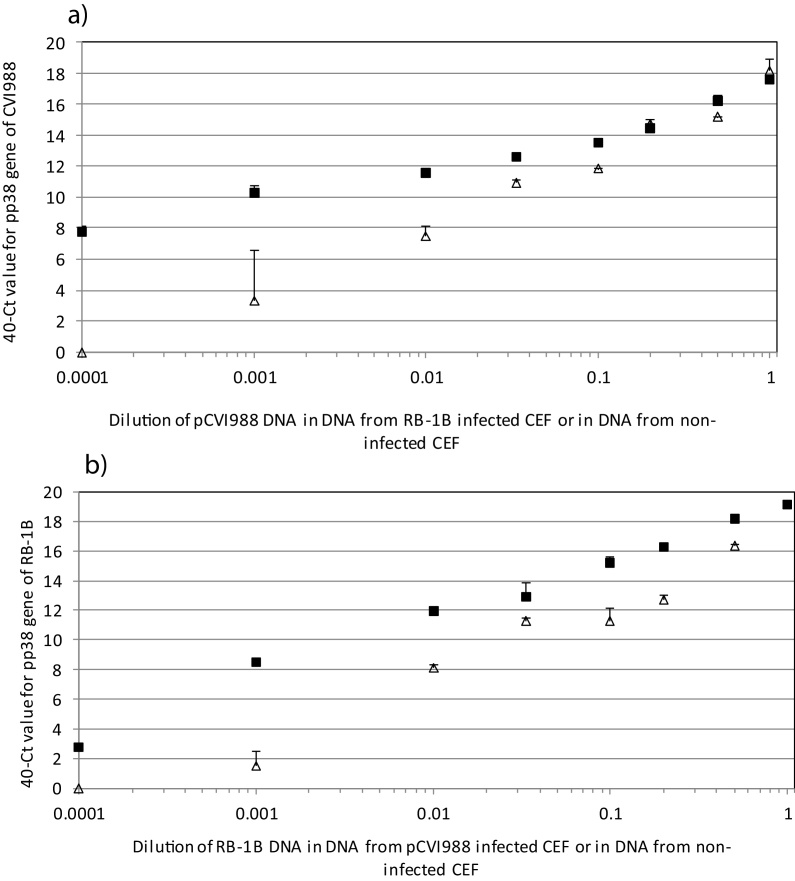
Quantitative accuracy of detection in virus mixtures using pp38 SNP q-PCR assays. DNA was prepared from CEF cells infected with pCVI988, and from CEF cells infected with RB-1B, and each was diluted to approximately 5 × 10^5^ virus genomes per 4 μl. (a) DNA of pCVI988-infected CEF was diluted in DNA of RB-1B-infected CEF (open triangles) or in DNA from non-infected CEF (black squares). (b) DNA of RB-1B-infected CEF was diluted in DNA of pCVI988 infected CEF (open triangles) or in DNA from non-infected CEF (black squares). Samples were run in q-PCR using either pp38-CVI probe or pp38-Vir(3) probe. The figures show mean 40-Ct values with standard error bars for duplicate dilution series and duplicate reactions (only upper error bars are shown, for clarity).

The BAC-specific q-PCR detected the same level of pCVI988 DNA, whether the pCVI988 DNA was diluted into RB-1B CEF DNA or into non-infected CEF DNA at each dilution (data not shown). The U_S_2-specific q-PCR detected the same level of RB-1B DNA, whether the RB-1B DNA was diluted into pCVI988CEF DNA or into non-infected CEF DNA at each dilution (data not shown). These assays confirmed that the dilution series were accurately prepared and directly comparable, and that there was no competition for reagents in these q-PCR assays. For the pp38-CVI q-PCR, 40-Ct values for pCVI988 DNA diluted in RB-1B CEF DNA were only slightly (≤1 Ct unit) lower than those for pCVI988 DNA diluted in non-infected CEF DNA, at dilutions down to 1:5 (Fig. 4a). At 1:10 and 1:30 the difference was greater (2Ct units) and statistically significant and, at dilutions of 1:10^2^, 1:10^3^, the difference was 4–7 Ct units (p < 0.001). Thus, at the greater dilutions, pCVI988 DNA could clearly be detected but not accurately quantified. At 1:10^4^ dilution in RB-1B DNA, the pCVI988 DNA was barely detectable, despite still being present at levels detectable by BAC q-PCR, showing that reagent competition in the pp38 SNP q-PCRs reduces detection of pCVI988 DNA when RB-1B DNA is in >5-fold excess. For the pp38-Vir(3) q-PCR, the effect of reagent competition on measured level of RB-1B was significant from 1:5 dilution in pCVI988 DNA (p < 0.01) and, although RB-1B DNA was still clearly detected at further dilutions, it would not be accurately quantifiable (Fig. 4b).

### 3.5 Validation of pp38 SNP q-PCR assays on chicken tissue samples

**Fig. 5:**
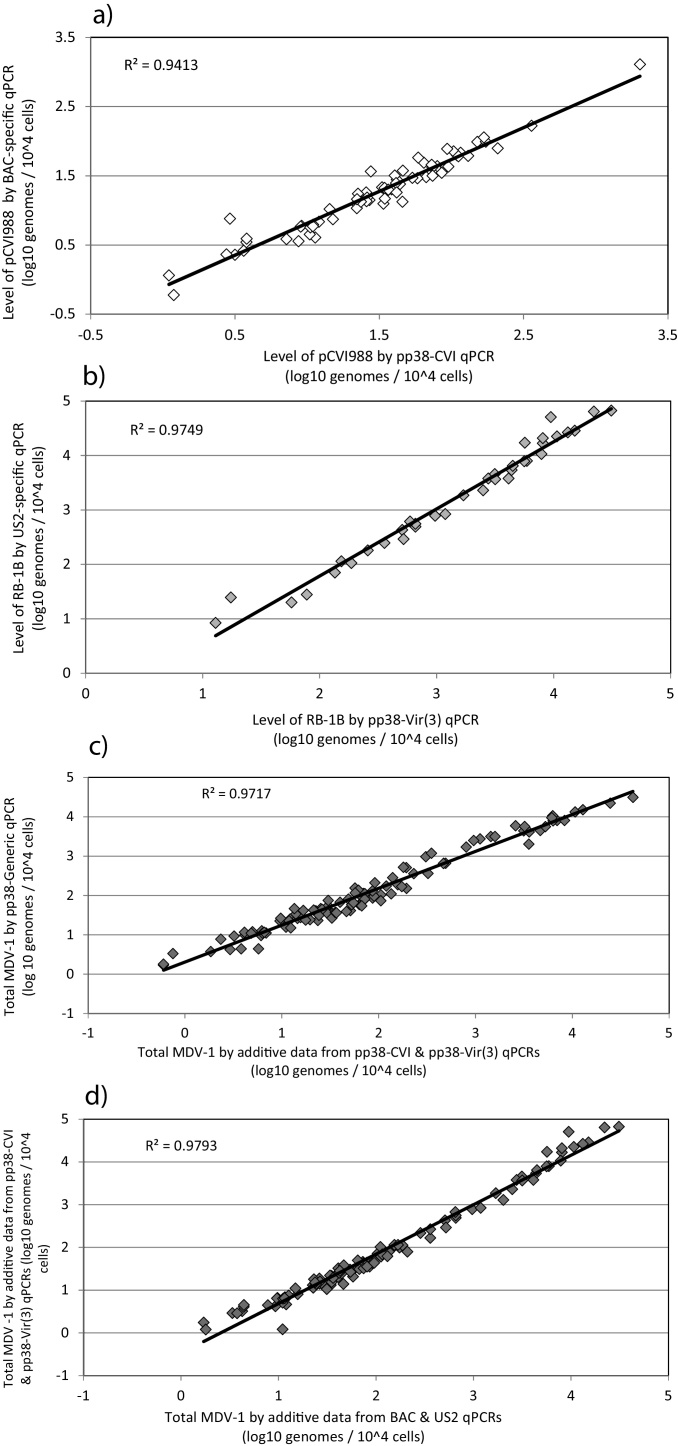
Validation of pp38 SNP q-PCR assays against BAC and U_S_2 q-PCR assays. Each data point shows values for an individual chicken (log_10_ genomes per 10^4^ cells). (a) Comparison of BAC q-PCR and pp38-CVI q-PCR to measure pCVI988 in vaccinated/non-challenged and vaccinated/challenged birds (n = 74). (b) Comparison of U_S_2 q-PCR and pp38-Vir(3) q-PCR to measure RB-1B in challenged/non-vaccinated and challenged/vaccinated birds (n = 79). (c) Comparison of pp38-Generic q-PCR with additive results from pp38-CVI and pp38-Vir(3) q-PCRs to measure total MDV-1 in all vaccinated and/or challenged birds (n = 102). (d) Comparison of additive results from pp38-CVI and pp38-Vir(3) q-PCRs, with additive results from BAC and U_S_2 q-PCRs to measure total MDV-1 in all vaccinated and/or challenged birds (n = 102).

Spleen DNA samples from experimental chickens were available from a previous study (Baigent et al., 2011) where the chickens were vaccinated at one day of age with BAC-cloned CVI988 virus (pCVI988) and/or challenged at 15 days of age with RB-1B, and four chickens per group sacrificed to collect spleen at each of eight time-points between 4 and 34 dpv (equivalent to −10 dpc to 20 dpc). These DNA samples were now subjected to pp38 SNP/*ovo* duplex q-PCR assays to determine level of vaccine virus and challenge virus as genome copies per 10^4^ cells. The calculated level of pCVI988 was compared with that previously determined using the BAC-specific q-PCR assay (Baigent et al., 2011), and the level of RB-1B was compared with that previously determined using the U_S_2-specific q-PCR assay (Baigent et al., 2011). The specificity of each of the reactions was as expected for the four groups of chickens: pCVI988 was detected only in the two vaccinated groups and RB-1B only in the two challenged groups. For each individual spleen DNA sample, total MDV-1 genome copy no., determined using the pp38-Generic probe, was compared with that obtained from the additive results from U_S_2 and BAC reactions, and from the additive results from pp38-CVI and pp38-Vir(3) reactions. Fig. 5 shows correlation between levels of pCVI988 vaccine virus, RB-1B challenge virus, and total MDV-1 measured by the two q-PCR systems. There was a highly significant relationship between the level of pCVI988 measured by the two q-PCR systems (p < 0.0001, Fig. 5a), and between the level of RB-1B measured by the two q-PCR systems (p < 0.0001, Fig. 5b). There was a highly significant relationship between the level of total MDV measured by pp38-Generic, BAC + U_S_2 and pp38-CVI + pp38-Vir(3) (p < 0.0001 for each of the comparisons; Fig. 5c and d). These data confirm that pp38-CVI and pp38-Vir(3) reactions show excellent specificity, and can be used to accurately quantify CVI988 vaccine and virulent MDV-1 in tissue samples containing both viruses in biologically relevant amounts.

### 3.6 Time-course of replication of commercial CVI988 vaccine, and RB-1B challenge virus in individual chicks

Having demonstrated, in the above experiment, that pp38 SNP q-PCR can be used to accurately quantify CVI988 vaccine and virulent MDV-1 in tissue samples containing both viruses in biologically relevant amounts, a further experiment was performed to examine replication of RB-1B challenge virus in experimental chickens vaccinated with one dose of commercial CVI988 vaccine. For each individual chicken, mortality and presence of MD lesions were recorded, and the level of CVI988 or RB-1B at each time-point was determined by pp38-CVI q-PCR and pp38-Vir q-PCR respectively. For each chicken, the detection kinetics of vaccine or challenge virus in PBL (Table 2) broadly correlated with detection kinetics in feather tips (data not shown). CVI988 was detected only in vaccinated chickens and RB-1B only in challenged chickens.

No death and no macroscopic MD lesions were observed in any chicken in Group A (vaccinated only). Each of these chickens was positive in pp38-CVI q-PCR and showed a uniform response, with high early replication of CVI988. RB-1B replicated to high levels in the PBL and feathers of non-vaccinated chickens (Group B). Ten out of 14 chickens (72%) developed the disease following RB-1B challenge, each of these having become RB-1B-positive by 13 dpc in PBL. Mortality occurred in two phases: ‘Early mortality’, from 8 to 11 dpc, was associated with rapid onset paralysis (6 chickens); and MD tumour phase, 20–33 dpc (4 chickens). At the end of the trial, three surviving chickens (#2421, #2436 and #2440) had high RB-1B levels and were observed to have small MD tumours; these chickens would likely have succumbed to MD had the trial continued for a further two weeks. The fourth survivor (#2342) did not become RB-1B-positive until 20 dpc, and had no visible lesions.

In Group C (vaccinated and challenged), one chicken (10%) reached humane end-point and was culled at 10 dpc. This bird was negative for CVI988 but had high early levels of RB-1B, indicating that it had not been successfully vaccinated. In the nine surviving chickens CVI988 was readily detected and no MD lesions were observed. In five of these chickens, RB-1B was not detected in either PBL or feather tips at any time-point. In the remaining four chickens, replication of RB-1B was considerably delayed, and was at a low level. Thus, vaccination had a major inhibitory effect on the replication of RB-1B in PBL and feathers of most chickens.

**Fig. 6:**
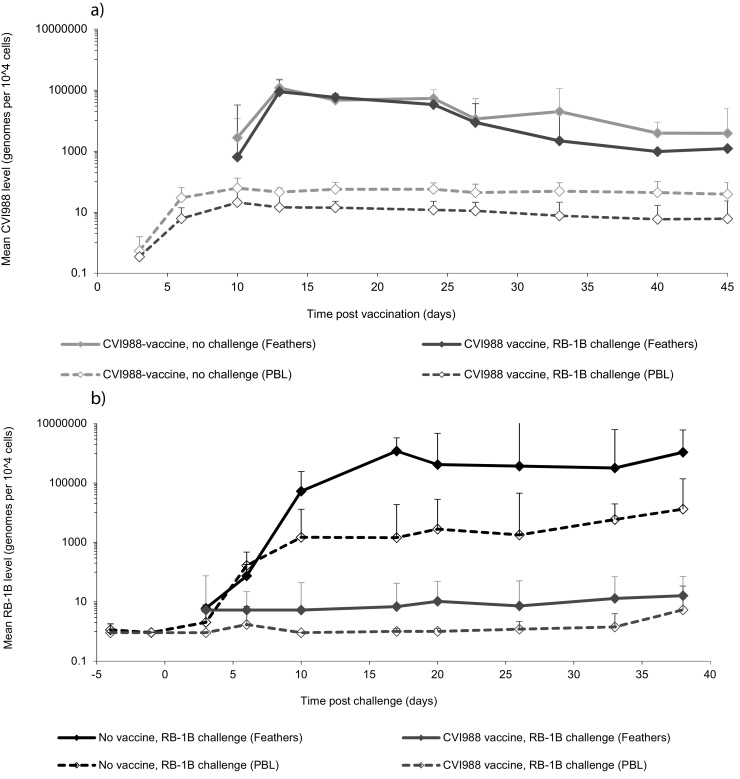
Time-course of replication of commercial CVI988 vaccine and RB-1B challenge virus in individual chickens. One-day-old Rhode Island Red chickens were divided between three experimental groups. Group A (n = 8) received CVI988 vaccine only, Group B (n = 14) received RB-1B challenge virus only, and Group C (n = 10) was both vaccinated and challenged. Feather and blood samples were collected from each chicken at regular intervals. DNA was subjected to pp38 SNP/*ovo* duplex q-PCR, using either pp38-CVI or pp38-Vir(3) as the pp38 detection probe, and the level of each virus quantified as genomes per 10^4^ cells. Mean virus levels (with 95% confidence intervals) for each group and each sample type at each time-point are shown for CVI988 vaccine virus (a) and RB-1B challenge virus (b). Values of 0.6 (baseline value) are considered negative. Only upper confidence intervals are shown for clarity.

The mean level of virus in feather tips and PBL was calculated for CVI988 (Fig. 6a) and RB-1B (Fig. 6b) for each group at each time-point. In feathers the mean level of CVI988 was lower in the RB-1B challenged group than in the non-challenged group from 33 dpv only, but the effect of RB-1B was not significant at any time-point. In PBL the mean level of CVI988 was lower in the RB-1B challenged group throughout the course of the experiment, this difference being significant from 17 dpv onwards (p < 0.05). Vaccination with CVI988 significantly lowered the mean level of measured RB-1B at 6 dpc and all time-points thereafter, in both PBL (p < 0.001) and feathers (p < 0.001).

## 4 Discussion

It has been a long-term goal to have a reliable test for differential quantification of CVI988 vaccine and virulent MDV-1 in chickens, since this is commercially important to confirm successful vaccination, to investigate causes of vaccine failure and to confirm a diagnosis of MD. To date, development of a q-PCR assay which is both accurately quantitative, and able to universally distinguish all virulent MDV strains from CVI988, has not been possible.

The current work resulted in development, optimisation and validation of an SNP-based real-time PCR assay which was specific, sensitive, efficient, reproducible, free from false-positive results, and enabled accurate differential quantification of CVI988 and American and European virulent MDV-1 strains, over a biologically relevant range of virus levels. The assay therefore offers several advantages over previously published differential assays.

It was necessary to identify both a suitable genetic difference on which to base the assays, and a suitable technology to enable quantitative differential detection of the difference. The selected genetic difference was a consistent SNP at position #320 in the MDV-1 pp38 gene, and the selected technology involved use of unconventionally short (15–17 nucleotide) TaqMan^®^ probes.

The pp38 gene sequences have now been determined for over 40 virulent isolates of MDV (NCBI database). The pp38 gene sequence is identical in all MDV-1 strains with the exception of bases #320 and #326 (Cui et al., 1991, Endoh et al., 1994, Spatz et al., 2007a, Spatz et al., 2007b), which differ between CVI988 and virulent strains. The polymorphisms result in a TspI restriction enzyme site in the pp38 gene of all MDV-1s except CVI988, and different antibody epitopes (Cui et al., 1999, Lee et al., 2005, Zhizhong et al., 2004), both of which may be used to differentiate CVI988 from virulent strains. At SNP #326, CVI988 has ‘G’ and the majority of virulent strains have ‘A’; however, some ‘atypical’ virulent strains (including GA strain, 571 strain, three Chinese strains and six recent Pennsylvanian field isolates) have ‘G’ and so SNP #326 cannot be used to reliably distinguish virulent strains from CVI988 (Gimeno et al., 2014). Conversely, SNP #320 is consistent between CVI988 (which has ‘G’ at this position) and all sequenced virulent strains (which have ‘A’) (Gimeno et al., 2014, Spatz et al., 2007a). Based on this polymorphism, Dunn et al., 2010, Dunn et al., 2012 developed pyrosequencing assays to distinguish and semi-quantify recombinant viruses (that had either the virulent-specific or CVI988-specific pp38 gene) in virus super-infection and competition experiments. Although assay specificity was 100%, sensitivity was relatively poor.

The current study therefore sought to investigate the possibility of using SNP #320 as a reliable biomarker for differential quantification of CVI988 and virulent MDV by real-time PCR. Real-time PCR technology for *differential detection* of SNPs is now widely used, but this only allows confirmation that the virus in question is present or absent, without quantification. The goal was achieved by using short TaqMan^®^ probes to differentially detect PCR products following amplification with common primers. Short probes can improve SNP detection by increasing quenching efficiency and specificity of PCR product detection. Probe pp38-Vir(3) also incorporates the ‘non-consistent’ SNP #326, in order to have a melting temperature similar to that of the pp38-CVI and pp38-Generic probes. This probe was chosen for use in the current work because (for all of the virulent MDVs tested here) it showed identical specificity, but greater sensitivity than the probe which contains only SNP #320. When these probes were first designed, atypical MDV field strains having ‘G’ at #326 had been observed only very rarely (Spatz et al., 2007a). However, since completion of these studies, several atypical strains have been identified in some regions of the USA (Gimeno et al., 2014) raising concerns that such strains are becoming increasingly prevalent. Using probe pp38-Vir(3) could therefore pose problems for the analysis of atypical field isolates: there is a possibility that such strains will not be efficiently detected by probe pp38-Vir(3) containing SNP #326 (whereas they would be efficiently detected using probe pp38-Vir(1) containing only SNP #320). The current work included one atypical MDV (strain 571) which was detected by both probes pp38-Vir(3) and pp38-Vir(1) with equal sensitivity, specificity and efficiency, which is reassuring. However, it will be important to test more atypical strains with both probes and, should issues with sensitivity, specificity and efficiency of probe pp38-Vir(3) be identified, it would be prudent to use only probe pp38-Vir(1) to test field samples in the future.

Simultaneous to the current work on developing this assay, Gimeno et al. (2014) sought to use the same polymorphism to achieve the same goal. However, their assay differs from the assay presented in this paper in terms of the molecular methods used to amplify and detect the polymorphism, their ability to fully validate the assay, and its quantitative accuracy. Specificity in real-time PCR is ideally required at two levels: PCR product specificity (differential amplification) and probe specificity (differential detection). The ‘Gimeno assay’ is based solely on differential amplification, while the current assay is based solely on differential detection. While both the current assay and the Gimeno assay have some complementary advantages and disadvantages, the former represents an improvement in that accurate differentiation and quantification has been validated over a biologically-relevant range of virus levels. Gimeno et al. (2014) developed a Mismatch Amplification Mutation Assay, which incorporates an intentional mismatch at the 3′ penultimate position in the PCR primers, a recognized method of increasing assay specificity. They used forward primers which incorporated the SNP at the last base (a CVI988-specific primer and a virulent MDV-specific primer) and a generic reverse primer, in a non-probe (SYBR-green) real-time PCR. PCR assays using primers designed to distinguish between a single nucleotide are frequently very inefficient. For this reason, the current work sought to develop probe-based TaqMan^®^ q-PCR using primers that would amplify pp38 of both CVI988 and virulent strains, and to incorporate the SNP into the short probes (a CVI988-specific probe and a virulent MDV-specific probe).

When either CVI988 only or virulent MDV only was present in a DNA sample, both the Gimeno assay and the current assay were highly specific and either virus could be accurately quantified over a wide dynamic range using standard curves. Both assays were reproducible and very sensitive but, while the limit of detection in the current assay was approximately 10 copies of virus genome, the Gimeno assay showed higher sensitivity detecting as little as one copy. Thus, both assays could successfully differentially detect CVI988 and virulent MDV.

However, both the current assay and the Gimeno assay had some limitations when attempting to specifically and accurately quantify CVI988 or virulent MDV in mixtures of virus-infected cell DNA (current assay) or DNA of plasmid carrying an insert of the pp38 gene of either CVI988 or virulent MDV (Gimeno et al., 2014) over a wide range of genome copies. The Gimeno assay suffered from ‘false positive’ results (loss of specificity if miss-matched sequence is in excess), and was only accurately quantitative if the homologous sequence was present above a certain level, and the heterologous sequence below a certain level. Conversely, the assay reported in this paper had issues with ‘false negative’ results (always specific, but loss of sensitivity if miss-matched sequence is in excess). This assay has an additional level of specificity through use of probes which each bound minimally to the miss-matched PCR product. However, use of universal primers to amplify both CVI988 and virulent MDV can yield false negatives since, in mixtures of CVI988 and virulent MDV DNA, these two target sequences will compete for primers. Consistent with this, when DNAs from CVI988 and virulent MDV were mixed over a wide range of ratios, competition for PCR primers by the predominant virus reduced the amplification (and thus detection) of the less abundant virus. Therefore, although the less abundant virus could still be detected, it could not be accurately quantified when the other virus was predominant.

Field samples will contain CVI988 and virulent MDV-1 in varying ratios. Both the current study, and Gimeno et al. (2014), validated the respective assays using tissue samples from experimental chickens which were either CVI988-vaccinated only, vvMDV-challenged only, or both CVI988-vaccinated and vvMDV-challenged. However, while Gimeno et al. (2014) used commercial CVI988 vaccine, the current study initially used BAC-cloned CVI988 vaccine which gave a significant advantage in confirmation of reliable and confident validation, since the BAC-sequence-specific/U_S_2-sequence-specific q-PCR system (Baigent et al., 2011), where there is no competition for primers, was used for validation of the pp38 SNP q-PCR assay. In the current study, use of the pp38-Generic reaction to validate the additive results of the virus-specific assays showed that, over a biologically-relevant range of CVI988 and virulent MDV levels generated in the *in vivo* challenge models used here, either virus could be quantified with a high level of accuracy. When CVI988-vaccination was performed correctly and effectively, the immune response kept the challenge virus at a level which was too low to impede accurate measurement of CVI988. Conversely, when CVI988 vaccination was not successful, the level of CVI988 was too low to impede accurate measurement of challenge virus.

The pp38 SNP q-PCR assay could therefore be used with confidence to follow kinetics of replication of commercial CVI988 and RB-1B in individual chickens. Mean kinetics of replication in PBL and feathers were consistent with previous reports for CVI988 in non-challenged chickens (Baigent et al., 2005b) and RB-1B in non-vaccinated chickens (Singh et al., 2010). Vaccination with CVI988 significantly reduced or almost completely inhibited detection of RB-1B in PBL and feathers, consistent with the observations of Islam et al., 2013, Islam et al., 2014 following challenge of CVI988-vaccinated chickens with an Australian vvMDV strain and distinction between the two viruses using the Australian strain-specific *meq* q-PCR assays (Renz et al., 2013). The reduction in virulent MDV load in CVI988-vaccinated chickens is greater than that observed in either SB-1 vaccinated chickens (Singh et al., 2010), HVT-vaccinated chickens (Islam et al., 2006b; Walkden-Brown et al., 2013a), or MDV-2/HVT bivalent-vaccinated chickens (Walkden-Brown et al., 2013a) consistent with the fact that CVI988 is the most effective vaccine. It was interesting that the mean level of CVI988 measured in PBL (but not in feathers) was significantly lower in the RB-1B challenged group than in the non-challenged group, whereas Islam et al. (2014) found that challenge on or after 5 dpv had little effect on CVI988 load. The possibility that this result in the current study could be a false result, reflecting competition for q-PCR reagents in samples containing both CVI988 and RB-1B reducing efficacy of CVI988 detection, was considered. However, given that the measured level of RB-1B was low in this group, it seems unlikely that such reagent competition had any marked effect on measurement of either CVI988 or RB-1B in these samples. Therefore, it is likely that challenge with RB-1B had a true effect on reducing replication of CVI988 under the experimental conditions used in the current study.

Prior to publication of validation of their assay by Gimeno et al. (2014), Haq et al. (2012) used this assay to examine replication of CVI988 vaccine and virulent challenge virus in feathers of experimental chickens. Consistent with the results of the current study, they observed that challenge with virulent MDV had no significant effect on replication of CVI988 in feathers, but that vaccination with CVI988 reduced the level of RB-1B measured in feathers. This reduction was more dramatic in the current study, but this may reflect differences in the vaccine used (Poulvac CVI988 vs Merial CVI988), the age at which vaccination was performed (day-old vs *in ovo*), or the fact that the Gimeno assay may yield false positive results.

The *in vivo* challenge model used in the current work (using chickens free of maternal antibody against MDV, a vaccination-challenge interval of 7 days, and vvMDV strain RB-1B as the challenge virus) did not result in any instance of the presence of relatively high levels of both vaccine and challenge virus together in an individual bird, and so there was no opportunity to demonstrate that the assay could accurately differentially quantify relatively high levels of co-infection in a chicken tissue sample. Use of a vv+ challenge strain and shorter vaccination-challenge interval might have resulted in higher levels of both viruses in the co-infected group. However, the challenge model was carefully chosen to aim for a balance between sufficient replication of challenge virus (to ensure that it still replicated in the CVI988-vaccinated group) and sufficient survival of chicks (to ensure multiple samples at multiple time-points for analysis). Inclusion of a challenged but non-vaccinated control group was necessary in order to observe the effect of vaccination on reduction of challenge virus load, but almost 50% of non-vaccinated chicks had died by 10 dpc. This early mortality would have been exacerbated had a shorter vaccination-challenge interval, and/or a vv+ challenge strain (Read et al., 2015) been used, so sufficient data would not have obtained from this control group, although the co-infected group would likely have had higher levels of both viruses. Use of chicks having maternal-antibody against MDV (to reduce the issues with early mortality), combined with a higher dose of RB-1B challenge virus, might provide higher levels of CVI988/RB-1B co-infection, and this would be interesting to examine.

The technology used in these MDV q-PCRs could be applied to other herpesvirus-induced diseases of man and animals, where live attenuated vaccines are used but there are minimal genetic differences between vaccine and virulent strains. Similar technology is available for human Varicella Zoster Virus, where SNPs in five genes identify four major subtypes and additional SNPs can differentiate the live attenuated Oka vaccine strain from wild-type strains (Quinlivan et al., 2005, Parker et al., 2006), but this was not quantitative.

Live attenuated vaccines, with high genetic similarity to virulent strains, are used against infectious laryngotracheitis virus (ILTV), another herpesvirus of chickens. Generation of new virulent ILTV strains by recombination between two attenuated vaccine strains was recently demonstrated in Australia (Lee et al., 2012). This potentially raises concerns for the long-term reliability of the assay presented in the current work for distinguishing CVI988 from virulent MDV. Given the now well-documented instability of the polymorphism at position 326 (Gimeno et al., 2014, Spatz et al., 2007a), how stable is the ‘consistent’ polymorphism at position 320 on which both this assay, and the Gimeno assay rely? Could mutation, or recombination between CVI988 and virulent strains, result in generation of new virulent viruses which have ‘G’ at position 320 and are therefore detected by the CVI988-specific assay and not by the virulent-specific assay? Although such viruses have been generated in the laboratory by genetic engineering (Cui et al., 1999, Lee et al., 2005) they have never been reported in the field: all virulent isolates sequenced to date, which have been isolated over a period of 40 years and cover the whole virulence spectrum, have ‘A’ at position 320. Therefore, this can be considered a highly stable polymorphism, so the pp38-Vir(1) probe should reliably differentiate virulent strains from CVI988 in the long-term. Nevertheless, should clinical signs of MD be observed in the absence of detection of any virus by the ‘virulent-specific’ assay, it would be prudent to sequence the whole virus genome and to perform *in vivo* pathogenicity studies in SPF chicks to further characterise the virus.

In summary, the pp38 SNP q-PCR assay allows fast, reliable, sensitive, specific and accurate differential quantification of CVI988 vaccine and virulent MDV in chicken tissue samples over a biologically-relevant range of virus levels, and thus represents an advance over currently available methods. In the commercial environment, the assays could be exploited for confirming success of CVI988 vaccination, for monitoring circulation of virulent field strains and for diagnosis of MD, using feather, blood or organ samples; and for monitoring MDV contamination of the poultry house environment in samples of poultry dust (Walkden-Brown et al., 2013b, Baigent et al., 2013). Ultimately, use of the pp38-CVI, pp38-Vir(1) and *ovo* probes in a single triplex reaction could be optimised to enable simultaneous quantification of CVI988 and virulent MDV.

## Figures and Tables

**Table 1:** q-PCR assay efficiency and reproducibility.

Primers	Probe	DNA template	Slope^a^	R^2^ value^b^	LOD^c^
pp38-FP + pp38-RP	pp38-CVI	CVI988-infected CEF DNA	3.52	0.995	5.7
pp38-FP + pp38-RP	pp38-Vir(1)	RB-1B-infected CEF DNA	3.53	0.995	29.6
pp38-FP + pp38-RP	pp38-Vir(3)	RB-1B-infected CEF DNA	3.48	0.994	9.6
pp38-FP + pp38-RP	pp38-Generic	CVI988-infected CEF DNA	3.27	0.992	8.9
pp38-FP + pp38-RP	pp38-Generic	RB-1B-infected CEF DNA	3.59	0.995	7.4
Ovo-FP + Ovo-RP	Ovo	Non-infected CEF	3.58	0.982	1.1

a measure of amplification efficiency (good efficiency is considered to be 90% [slope = 3.59] − 105% [slope = 3.21]).

b R^2^ coefficient of determination, a measure of linearity.

c Limit of detection (copies of target gene).

**Table 2:** Measurement of CVI988 vaccine and RB-1B challenge virus in PBL of experimental chickens, using pp38 SNP q-PCR assay.

Group	Bird #	Virus	Sampling time-point: PBL (virus level: genome copies per 10,000 cells)										Survived S/Died D (time of death dpc)
			3 dpv	6 dpv	10 dpv	13 dpv	17 dpv	24 dpv	27 dpv	33 dpv	40 dpv	45 dpv	
			−4 dpc	−1 dpc	3 dpc	6 dpc	10 dpc	17 dpc	20 dpc	26 dpc	33 dpc	38 dpc	
A Vacc only (level of CVI) a	2428	CVI	—c	66	81	43	101	85	79	135	144	ND^d^	S
2433	CVI	–	7	36	31	40	21	15	23	22	18	S
2434	CVI	–	74	75	53	75	57	56	42	10	31	S
2437	CVI	12	84	110	78	69	93	105	115	137	134	S
2442	CVI	–	12	77	48	45	54	42	38	54	47	S
2444	CVI	–	23	61	29	29	44	31	37	40	35	S
2446	CVI	–	42	9	ND	24	34	15	16	15	9	S
2447	CVI	–	17	192	52	151	129	91	89	92	113	S

B Chall only (level of RB-1B) b	2401	RB1B	–	–	3.1	913	13231	15924	30017	D^e^	D	D	D 20 dpc
2403	RB1B	–	–	–	133	3138	D	D	D	D	D	D 11 dpc
2405	RB1B	–	–	–	249	D	D	D	D	D	D	D 8 dpc
2421	RB1B	–	–	–	336	6586	2968	3783	7878	16290	32241	S
2424	RB1B	–	–	–	309	D	D	D	D	D	D	D 10 dpc
2429	RB1B	–	–	57	1421	D	D	D	D	D	D	D 8 dpc
2432	RB1B	–	–	–	–	–	–	4	17	1270	–	S
2435	RB1B	–	–	–	35	1528	1739	2632	3491	9352	D	D 33 dpc
2436	RB1B	–	–	–	73	2171	2881	5375	5391	4592	4902	S
2439	RB1B	–	1.1	2.8	190	3631	4170	8707	D	D	D	D 24dpc
2440	RB1B	–	–	–	99	4400	2532	3893	6842	7983	14220	S
2443	RB1B	–	–	4.0	225	2493	8329	16123	D	D	D	D 21 dpc
2445	RB1B	–	–	23	697	D	D	D	D	D	D	D 8 dpc
2448	RB1B	–	–	–	419	D	D	D	D	D	D	D 8 dpc

C Vacc & Chall (level of CVI and level of RB-1B)	2404	CVI	–	10	98	31	18	9	12	12	9	7	S
	RB1B	–	–	–	–	–	–	–	–	–	87	
2407	CVI	–	12	44	22	7	12	11	10	11	21	S
	RB1B	–	–	–	–	–	–	–	–	–	–	
2414	CVI	–	12	53	26	34	32	28	41	50	108	S
	RB1B	–	–	–	–	–	–	–	–	–	–	
2417	CVI	–	6	25	4	5	2	2	0.3	0.3	1	S
	RB1B	–	–	–	–	–	–	–	–	–	–	
2418	CVI	–	3	28	33	24	29	25	21	15	24	S
	RB1B	–	–	–	–	–	–	–	–	–	–	
2419	CVI	–	6	7	10	11	8	16	9	7	12	S
	RB1B	–	–	–	–	–	–	–	–	–	–	
2420	CVI	–	–	–	–	D	D	D	D	D	D	D 10 dpc
	RB1B	–	0.9	0.9	460	D	D	D	D	D	D	
2422	CVI	–	23	37	23	10	10	11	9	4	2	S
	RB1B	–	–	–	–	–	2	2	10	50	134	
2423	CVI	–	6	20	31	17	10	12	7	4	0.3	S
	RB1B	–	–	–	–	–	–	–	–	–	171	
2425	CVI	–	9	51	65	22	25	8	4	3	4	S
	RB1B	–	–	–	–	–	–	–	–	–	3	

a All Group A birds were negative for RB-1B at each time-point (data not shown).

b All Group B birds were negative for CVI988 at each time-point (data not shown).

c —indicates samples that were considered negative in the q-PCR (40-Ct value ≤1) and resulted in a baseline value of 0.6 genome copies per 10^4^ cells.

d ND = Not done (chicken inadvertently not sampled at this time-point).

e D = bird dead, therefore no sample at this time-point.
